# Exploiting Rye in Wheat Quality Breeding: The Case of Arabinoxylan Content

**DOI:** 10.3390/plants12040737

**Published:** 2023-02-07

**Authors:** Maria Chiara Piro, Hilde Muylle, Geert Haesaert

**Affiliations:** 1 Department of Plants and Crops, Faculty of Bioscience Engineering, Ghent University, Valentin Vaerwyckweg 1, 9000 Ghent, Belgium; 2 Plant Sciences Unit, Flanders Research Institute for Agriculture, Fisheries and Food (ILVO), Caritasstraat 39, 9090 Melle, Belgium

**Keywords:** arabinoxylan, alien chromatin, introgression breeding

## Abstract

Rye (*Secale cereale* subsp. *cereale* L.) has long been exploited as a valuable alternative genetic resource in wheat (*Triticum aestivum* L.) breeding. Indeed, the introgression of rye genetic material led to significant breakthroughs in the improvement of disease and pest resistance of wheat, as well as a few agronomic traits. While such traits remain a high priority in cereal breeding, nutritional aspects of grain crops are coming under the spotlight as consumers become more conscious about their dietary choices and the food industry strives to offer food options that meet their demands. To address this new challenge, wheat breeding can once again turn to rye to look for additional genetic variation. A nutritional aspect that can potentially greatly benefit from the introgression of rye genetic material is the dietary fibre content of flour. In fact, rye is richer in dietary fibre than wheat, especially in terms of arabinoxylan content. Arabinoxylan is a major dietary fibre component in wheat and rye endosperm flours, and it is associated with a variety of health benefits, including normalisation of glycaemic levels and promotion of the gut microbiota. Thus, it is a valuable addition to the human diet, and it can represent a novel target for wheat–rye introgression breeding.

## 1. Rye, an Ally to Wheat Breeding

The endeavour of combining the genetic material of rye (*S. cereale* subsp. *cereale* L.) and wheat (*T. aestivum* L.) has a long history. The initial interest towards wheat–rye hybridisation resided in the possibility to combine the yield potential and technological qualities of wheat with the adaptability and resilience of rye. The first recorded attempts at wheat–rye hybridisation date back to the 19th century, and it was in 1875 that Scottish agronomist A. S. Wilson reported the very first viable, but infertile, wheat–rye hybrid plant [1]. Eventually, these breeding efforts even gave rise to an entirely new crop: the man-made hybrid cereal triticale (×*Triticosecale* Wittm.) [1]. In triticale, the entire chromosome set of rye (RR) is combined with one (AA), two (AABB) or all three (AABBDD) chromosome sets of wheat, resulting in tetraploid, hexaploid, and octoploid triticale, respectively [1]. Nevertheless, only hexaploid triticale (AABBRR) is a successfully established crop, whereas the other forms find application as breeding material [1].

Of course, whole-genome introgression such as in the case of triticale has implications that go beyond the scope of improving a single or a few specific traits of interest in wheat itself. Thus, introgressions from rye to wheat at sub-genome scale, in the form of single-chromosome substitution, or translocation of chromosome segments have also been widely researched in wheat breeding since the 1920s [2]. The main goal has mostly been the enhancement of wheat resistance to pests and diseases, and to this end, the 1RS, 2RL, 3RS, 4RS, 4RL, and 6RL rye chromosome segments have been introgressed in the wheat background [3]. Among these, the introgression of the 1RS segment, in the form of 1RS.1BL or 1RS.1AL translocation, was the first to be achieved, and the one that undoubtedly had the greatest agricultural impact [2,3]. Loci for resistance to leaf, stem, and stripe rust, and to powdery mildew were introduced by means of this translocation in hundreds of wheat cultivars released between the 1960s and 1990s [3]. The success of this translocation lasted even after these resistances were broken [2]. This continued success is likely to be attributed to a locus for root biomass present on the 1RS segment, which positively affected the yield of the winter wheat varieties carrying the translocation [2,4].

Clearly then, it can be worthwhile to investigate rye chromatin not only for the presence of loci related to disease and pest resistance. For example, genetic studies on triticale already evidenced the contribution of its R genome to several other traits of agronomic interest, such as aluminium tolerance, biomass yield, and allelopathic effects [5,6,7]. Nevertheless, wheat grain and end-use quality traits were never considered as traits that could profit from the introgression of rye chromatin. Rather, until now, rye chromatin has been viewed as detrimental to wheat quality. For example, a crucial issue that quickly emerged with the 1RS.1BL or 1RS.1AL translocations was the concomitant introgression in the wheat background of a major rye storage protein locus—*Sec-*1—which encodes for γ-and ω-secalins [8]. At the same time, gliadin loci and *Glu-*3 loci encoding for low-molecular-weight glutenin subunits are lost together with the 1BS or 1AS arm, resulting in an overall reduction in end-use quality, with 1RS.1BL wheats suffering from the most severe quality drawbacks [9,10]. Nevertheless, “quality” does not refer only to the suitability for a certain end-use—such as, for example, baking, in the case of wheat—but also to nutritional quality. In recent years, nutritional aspects of wheat-based products have been gaining more and more popularity among consumers, and the wheat industry is adapting to this trend as well [11,12]. It is in the context of breeding for wheat nutritional qualities that rye chromatin can once more find a valuable and innovative application; thanks to the higher contents of dietary fibre (DF) components and bioactive compounds, such as folates or sterols, of rye [13]. Thus, in order to illustrate the potential application of rye chromatin introgression in wheat quality breeding, DF content of wheat white flour is taken as a case study in the present work. Special attention is therefore dedicated to the main structural and functional features of the major DF component of white flour—arabinoxylan—and to the current understanding of the genetic basis of arabinoxylan content in both rye and wheat.

## 2. A Case Study for Wheat Quality Breeding: Dietary Fibre Content

**Figure 1:**
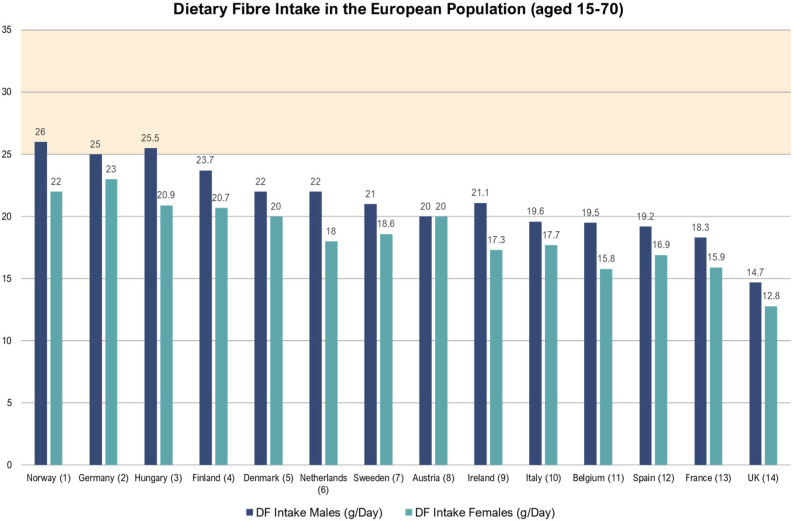
The daily dietary fibre intake recommended by the WHO (area shaded in yellow) is hardly met by the European adult population. This requirement is reached only in a minority of countries (i.e., Norway, Germany, and Hungary), and just by the male segment of the adult population [18,19,20,21,22,23,24,25,26,27,28,29,30,31] (Data aggregated by [14]).

DF content is a nutritional aspect that has been receiving an ever-increasing attention in the cereal food industry, due to the rich variety of health benefits that are associated with an adequate DF intake [11,14]. Nevertheless, by taking the European adult population as an example, it is obvious that the recommended DF daily intake of at least 25 g/day set by the WHO is still far from being reached. [14,15] (Figure 1).

Cereal-based products constitute the major source of DF in the diets of many European countries, providing, for example, about 33% of DF in Belgium and Spain, and up to 49% of total DF in Sweden [14]. Wheat white flour is often a main ingredient of such products. Conventional milling to yield white flour separates the starchy endosperm from the fibre-rich bran layers, thus reducing considerably the DF provision of white flour-based foods. Indeed, DF content amounts to only about 3% of the total dry matter of wheat white flour, whereas it accounts for up to 15% of wheat wholegrain flour dry mass [16,17].

The principal constituent of wheat white flour DF is the cell wall polysaccharide arabinoxylan (AX), where it accounts for 60–70% of total DF [16,17,32]. Like other sources of DF, AX is considered a health-promoting compound in the context of the human diet. Indeed, AX has prebiotic qualities (i.e., resistance to gastrointestinal absorption and hydrolysis, fermentation by the gut microbiota, and promotion of colonic *Bifidobacterium* spp. growth) [33]. Moreover, the addition of AX to the human diet positively contributes to important metabolic processes, such as the normalisation of postprandial glucose levels, and the reduction of blood triglycerides and cholesterol [33]. Enhancing wheat AX content to improve white flour DF provision is becoming an established goal in the European wheat breeding work frame. The European HEALTHGRAIN [34] programme delivered promising results in this direction. In fact, the screening highlighted significant variability in the AX content of winter wheat—with AX content ranging from 1.35% to 2.75% of white flour dry matter [35]. Several studies were also dedicated to understanding the genetic and environmental contributions to the variation of AX content in wheat wholegrain and white flours [11,36,37,38]. These studies all agreed that there is a significant environmental effect on wheat AX content, but also that the genotypic effect is sufficient to make breeding for higher AX a feasible objective [11,36,37,38]. Indeed, the estimated heritability for this trait is 72% [17].

In the context of this breeding effort, rye has considerable potential as an alternative genetic resource. In fact, AX is the principal cell wall polysaccharide also in the rye endosperm and, therefore, the main source of DF in rye flour as well [13,39]. In terms of AX content, rye is known to be richer in AX compared to wheat—with rye flour AX content ranging from 3.11% to 4.31% of dry matter in the HEALTHGRAIN diversity screen [13]. Then, similarly to the case of wheat, research on rye wholemeal highlighted a significant genotypic effect on AX content, and a rather high estimated heritability ranging from 82% to 89% [40,41]. A proof of concept of the increasing effect of rye genetic material on wheat flour AX content can be readily found in triticale—the hybrid progeny of wheat and rye [42]. Although no comprehensive genetic study is yet available on the specific contribution of triticale’s R genome to its AX content, the AX content of triticale was shown to be intermediate to that of wheat and rye, and to also show inter-cultivar variation [42,43,44].The reported contents of AX in triticale range from the equivalent to up to about 20% higher than those found in wheat [42,43,44]. To our knowledge, instances of triticale varieties with an AX content equivalent to that of rye have not yet been reported in literature. This may depend on the rye parentage or on the genomic composition of the triticale variety, as speculated already for “Armadillo” triticale types, which are known to possess chromosome 2D of wheat in place of the corresponding rye chromosome [43,45]. The importance of the choice of the rye chromatin source is further stressed by recent works on wheat–rye single chromosome addition lines, which also yielded promising results for this novel application of rye chromatin in wheat quality breeding [46,47,48]. Namely, the addition of chromosomes 1R, 4R, and 6R lead to a significant increase in AX content in the disomic addition lines, compared to the wheat parent [46,47,48]. In the case of the addition of chromosome 1R, the AX content increased up to the level of the rye parent [47]. Such research not only supports the feasibility of this breeding strategy, but also suggests more specific introgression targets already.

## 3. The Main Features of Arabinoxylan in Rye and Wheat

### 3.1. General Structure and Characteristics

AX is made up of a backbone of (1-4)-linked β-D-xylopyranosyl (Xyl*p*) residues, which can be substituted by α-L-arabinofuranosyl (Ara*f*) residues on the O-2, O-3, or both positions [49]. In rye and wheat, the most common substitution is the O-3 mono-substitution, followed by O-2-O-3 di-substitution, whereas the O-2 mono-substitution is rare [49,50,51]. Rye AX generally displays a higher percentage of mono- and di-substituted Xyl*p* compared to wheat AX [49,50,51]. Indeed, the Ara*f* to Xyl*p* ratio of rye flour ranges from 0.66 to 0.76, whereas that of wheat flour ranges from 0.50 to 0.70, indicating a higher degree of substitution in rye AX compared to wheat AX [13,35]. The O-3-linked Ara*f* residues can be substituted on the O-5 position by the hydrocinnamic acids ferulic acid and *p*-coumaric acid [49]. Ferulic acid, in turn, can be subject to oxidative cross-link, yielding diferulate bridges between adjacent AX chains [49]. The degree of diferulate cross-linking together with the degree of substitution with Araf residues affect the solubility of AX, so that AX is usually distinguished into two fractions: water-extractable AX (WE-AX) and water-unextractable AX (WU-AX) [49]. Oxidative cross-linking can occur not only between two ferulic acid residues, but also between ferulic acid and tyrosine [52]. Thus, a fraction of AX can be covalently bound to proteins in wheat and rye kernels. [52,53]. These covalent AX-protein interactions resist thermal and protease treatment, as shown by the persistence of protein in AX isolates of rye and wheat wholemeal [53]. Rye WE-AX and WU-AX isolates display a protein content of 8.4% and 6.1%, respectively, whereas wheat WE-AX and WU-AX isolates are characterised by a higher protein content of 13.0% and 12.2%, respectively [53]. Thus, AX is likely to be linked to proteins in the rye kernels to a lesser extent as compared to wheat.

### 3.2. Grain Localisation and Functionality

AX is localised primarily in the cell walls of various tissues of the rye and wheat grains as shown by different staining techniques. Images taken after xylanase probe staining show a bright signal originating from the nucellar epidermis and aleurone cell walls [54]. Subaleurone cell walls are distinctly outlined in both rye and wheat grains, whereas the central endosperm cell walls appear more intensely labelled in wheat than in rye [54]. Immunofluorescence analysis at various stages of development of the wheat grain showed that the deposition of AX in the cell walls is a gradual and dynamic process [55]. At 8 days after anthesis (DAA), only the nucellar epidermis and nucellar projection most proximal to the endosperm were labelled by the antibody probe. From 12 DAA, the aleurone and endosperm cell walls also became visible, with strong labelling around the crease region and weaker labelling extending radially from there. Finally, at 28 DAA, the cell walls of the entire endosperm tissue could be distinctly labelled [55]. Recent research on wheat showed that AX is also found in intracellular vesicles in various grain regions: in the apoplast between the nucellar epidermis and the endosperm tissues, and in the endosperm cavity [56]. The endosperm cavity separates the nucellar projection from the endosperm transfer cells and it is filled by a viscous, gel-like substance that persists even after desiccation [56,57]. This substance is made of various compounds, including lowly Ara*f*-substituted AX, hydrocinnamic acids (i.e., ferulic acid and *p*-coumaric acid), and ferulic acid dimers [56,58]. The presence of AX and hydrocinnamic acids is likely responsible for its gel quality and high hydration level [56]. Indeed, both a low degree of Ara*f* substitution and a high content of ferulic acid favour AX gelling capacity [59,60]. Furthermore, films of lowly Ara*f*-substituted AX show improved water absorption [61].

As it is the major cell wall polymer of the endosperm cell walls, AX constitutes a fundamental structural component of the rye and wheat grains. However, its functionality seems to extend to more than being the main cell wall building block. AX, with its high water-binding capacity, is instrumental in determining the high hydration level of the endosperm cavity sap, which is maintained throughout grain development [56]. Furthermore, the increased thickness of wheat aleurone cell walls, and consequently higher AX, are associated with greater water mobility within the seed and reduced water inward absorption and outer diffusion [62,63]. Thus, at least in wheat, AX appears to be a major regulator of the grain hydration state throughout development and after desiccation. To our knowledge, similar studies on the relationship between AX and water mobility within the rye grain are not yet available.

### 3.3. Variation of Arabinoxylan Content and Composition in Response to Environmental Conditions

The degree of substitution of rye and wheat AX is not only dynamic throughout seed development, but it is affected by environmental conditions as well [64,65]. In particular, the available literature indicates that both AX content and AX structure are highly responsive to water availability and ambient temperature. Indeed, the WE-AX content of wheat appears to be positively correlated with higher rainfall during grain filling [66]. Likewise, rye WE-AX content was found to be positively correlated with precipitation and, at the same time, negatively correlated with the mean temperature registered from heading to harvest [67]. It must be noted, however, that these correlations between WE-AX and wet, cool conditions may be partly biased by the higher xylanase activity in the grain observed in such environmental regimes [66].

Opposite conditions, meaning higher temperatures and low water availability, also affect AX content and composition [64,65]. In fact, there is a significant increase in the ratio of mono- to di-substituted Xyl*p* residues of wheat AX with temperatures up to 30 °C combined with water scarcity during grain filling [64,65]. Similarly, the proportion of unsubstituted AX oligosaccharides increases under heat, drought, and combined heat and drought stress (i.e., 35/20 °C and/or 40–45% soil moisture; [68]). Such a response implies a lower fraction of WE-AX in the mature grain in hot and dry conditions. In line with this result, Rakszegi et al. [68] found that the WE-AX content of three winter wheat varieties was reduced under drought, or combined drought and heat stress. Conversely, heat stress alone determined an increase in the WE-AX content of all three varieties [68]. Nevertheless, this latter result is in accordance with previous literature on spring wheat. Zhang et al. [69] also observed a significant increase in WE-AX content under heat stress alone in the spring wheat cultivar “AC Crystal”. Therefore, more than one regulatory mechanism may affect AX content and composition of wheat under different combinations of water availability and ambient temperature.

A relationship between AX content and drought was recently highlighted in rye as well [41]. An average 25% increase in TOT-AX content and an average 47% increase in WE-AX content of wholemeal rye flour were observed under natural drought conditions [41]. This modulation of the AX content appears to be linked to a general increase in sink activity of the rye grain. In fact, despite a grain yield decrease of 8% under drought conditions, no reduction of starch content or thousand kernel weight was observed in the studied rye population [41]. Rather, the average starch content was stable, whereas there was an average 20% increase in thousand kernel weight [41]. Thus, the modulation of AX content in response to natural drought seems to not impair, but rather to sustain, the grain filling process in rye.

## 4. The Genetic Basis of Arabinoxylan Content

### 4.1. Studies in Rye

Investigations about the genetic basis of rye AX content were carried out both on cultivated rye (*S. cereale* subsp. *cereale* L.) and the hybrid rye *S. cereanum* (*S. cereale* L. × *S. montanum* Guss., syn. *S. strictum* subsp. *strictum* Presl.). Until very recently, all the knowledge on this topic was derived from the characterisation of wheat–rye translocation and addition lines (Table 1).

Among these studies, the only one that examined the entire rye genome was carried out by Boros et al. [70], already 20 years ago. In their work, Boros et al., exploited non-commercial material, which included wheat–rye disomic addition lines (*T. aestivum* cv “Chinese Spring” × *S. cereale* cv. “Imperial”, “Blanco”, and “King II”; *T. aestivum* line BH1146 × *S. cereale* cv. “Blanco”), single disomic D-genome substitutions in hexaploid triticale (cv. “Presto”, “Rhino”), and homozygous centric wheat–rye translocations 2RS/2BL, 2BS/2RL, 3DS/3RL, and 3RS/3DL in cv. “Chinese Spring”. Using this material, Boros et al. determined the presence of AX-related loci on chromosomes 2R, 5R, and 6R. Indeed, the respective addition lines and both the 2RS/2BL and the 2BS/2RL centric wheat–rye translocation lines showed significantly higher DF and AX content; whereas the corresponding D-substituted triticales showed a reduction in DF and AX content, except for the 2R D-substituted triticale [70]. Contradicting results were obtained between the addition of whole 3R and 7R chromosomes, and the 3RL translocation and 7RL ditelosomic addition. In fact, the addition of these chromosomes in their entirety proved detrimental to DF and AX content, whereas the translocation or addition of only the respective long arm determined an increase in DF and AX content [70]. Boros et al. suggested the possible presence of antagonistic loci on chromosome 7R, however, to our knowledge, no other study investigated these findings in more detail. Boros et al. observed a reduction of DF content also in the 1R addition line; on the contrary, Szakács et al. [47] found their 1BL/1RS translocation line (*T. aestivum* line “Mv9kr1” × *S. cereanum* cv. “Kriszta”) to have significantly higher AX content. Similarly, Schneider et al. [46] also observed increased AX in the 1R disomic addition line derived from the same cross.

With the new development of high-quality genome assemblies for rye, it is now possible to adopt-*omic* approaches to dissect the genetic basis of a complex quantitative trait such as rye AX content [71,72]. The first of such studies are a transcriptomic investigation by Kozlova et al. [73] and a GWAS by Siekmann et al. [41]. The work of Kozlova et al. looked specifically for genes that are differentially expressed in rye kernels at milk, dough, and full kernel maturity developmental stage [74]. The corresponding predicted protein sequences were then assigned to known AX-related protein families based on the presence of characteristic protein domains [73]. This approach allowed for the identification of several candidate genes for AX biosynthesis in rye (Table 2).

The most highly expressed candidates were Secce7Rv1G0509920 for *IRX14*, Secce7Rv1G0479050 for *IRX9*, Secce3Rv1G0209270 and Secce3Rv1G0209200 for *IRX10*, Secce6Rv1G0408940 for *XAT1*, and Secce3Rv1G0161850 for *BAHD01* [73]. Performing a simple BLAST search of these gene models against the wheat genome assembly (Chinese Spring IGWS RefSeq v2.1; [75]) reveals their homology with the main AX biosynthetic genes of wheat. Notably, there is not any overlap between these candidate genes and the protein-encoding genes found to be associated with TOT-AX and WE-AX content in the GWAS by Siekmann et al. [41]. In this study, Siekmann et al. found a total of 416 and 433 SNPs to be associated with TOT-AX and WE-AX content, respectively. Of these SNPs, 31 and 15 were consistently associated with TOT-AX and WE-AX content across the eight different environments examined in the study [41]. The corresponding protein-encoding genes belong to a variety of protein families, including xylosyl- and glycosyl-transferases, which might be involved in the dynamic restructuring of rye AX in response to environmental stress, rather than the main biosynthetic process [41].

### 4.2. Studies in Wheat

A larger body of literature is available on the genetic control of wheat AX. Nevertheless, only few of the genes involved in the biosynthetic pathway of AX chains are currently characterised. Three genes, *TaIRX9b*, *TaIRX14*, and *TaIRX10*, encode the enzymes necessary for the assembly of the Xylpyranosyl (Xyl*p*) residues into the xylan backbone of AX [76,77,78,79]. *TaIRX9b* (transcript TaGT43_2, GenBank/EMBL accession no AM701827.1; [76,78]) and *TaIRX14* (transcript TaGT43-4, GenBank/EMBL accession no HM236487.1; [79]) both encode a glycosyltransferase 43 (GT43) family member. *TaIRX10*, instead, encodes a GT47 family member. Two transcripts have been described for this third gene, namely *TaGT47-13* (GenBank/EMBL accession no HM2364851.1; [79]) and *TaGT47_2* (GenBank/EMBL accession no AM698096.1; [78]). *TaIRX10/TaGT47-13* appears to be expressed in the outer grain tissues, whereas *TaIRX10/TaGT47_2* is almost exclusively expressed in the endosperm [78].

Three more genes are responsible for the decoration of the xylan chain. *TaXAT1* (GenBank/EMBL accession no FR846232.1) is the only gene involved in the addition of arabinofuranosyl (Ara*f*) residues to the xylan backbone that was described in wheat so far [17,80]. This gene encodes a GT61 family member that adds the Ara*f* residues at position 3, resulting in monosubstituted Xyl*p* [80]. At least three more genes are supposedly needed: two for the Ara*f* substitution at positions two and three in disubstituted Xyl*p*, and one for the feruloyl-Ara*f* substitution in monosubstituted Xyl*p* [17]. Then, the *TaBAHD02* and *TaPRX19D* genes were recently proposed to be responsible for the addition of ferulic acid (FA) residues and for diferulate crosslinking, respectively [76]. The *TaBAHD02* genes belong to the plant BAHD gene clade and encode acyl-CoA-utilising enzymes; in wheat, these genes are found arranged in tandem on every sub-genome [76,81]. BAHD genes have already been linked to the addition of FA and *p*-coumaric acid (*p*CA) to AX in model grasses. In *Setaria viridis* (L.), the suppression of *SvBAHD05* results in a 50% decrease in *p*CA-Ara*f*, whereas silencing of *SvBAHD01* leads to a 60% decrease in AX feruloylation [82,83]. In *Brachypodium distachyon* (L.), methyl-jasmonate treatment triggers the up-regulation of BAHD genes together with an increase of AX-linked *p*CA and FA [84]. Similarly, in wheat *TaIRX9b* triple mutants, the up-regulation of the *TaBAHD02* paralogues is accompanied by a two-fold increase in FA content [76]. The *TaPRX19D* gene encodes a class III peroxidase. Peroxidases of this family are already known to mediate oxidative cross-linking of FA [85]. In the wheat *TaIRX9b* triple knock-out mutants, all *TaPRX19D* homeologues are upregulated, followed by a 2.6 increase in diferulate content [76].

In addition to the aforementioned genes, there are also several quantitative trait loci (QTLs) that are known to affect AX content in wheat (Table 3).

Various candidate genes have been identified within some of these QTL regions. Charmet et al. [86] investigated 13 candidates among the genes located within the QTL regions mapped by Martinant et al. [87] and Groos et al. [88] on chromosomes 1B and 7A, their homoeologues, and the genes annotated as glucosyltransferase. Only one gene located on chromosome 7AL showed polymorphism significantly associated with variation in WE-AX A/X ratio and in WE-AX viscosity [86]. This gene encodes a caffeic acid O-methyltransferase and it is involved in the conversion of caffeic acid to FA [86,89]. Its location falls within a QTL region associated with dough viscosity ascribed to soluble pentosans [88]. Quraishi et al. [90] also selected three genes out of 73 differentially expressed candidates based on their co-location with the QTLs on chromosomes 3A, 3D, 6B, and 1B found in their study. These three genes encode a kinase inhibitor, co-located with the 3A and 3D homoeologous QTLs, a translation initiation factor, co-located with the 6B QTL, and a ribosomal protein co-located with the 1B QTL [90]. Their orthologous counterparts in *B. distachyon* are the genes Bradi2g62760.1, Bradi2g17180.1, and Bradi4g30780.1, respectively. Finally, Quraishi et al. [90] selected four more genes exploiting a synteny approach. The genes were selected among 44 orthologous candidates from *B. distachyon*, rice (*Oryza sativa* L.), and sorghum (*Sorghum bicolor* L.) that are co-linear with the 1B QTL identified in their study [90]. These genes supposedly encode an anthocyanidin 5,3-O-glucosyltransferase (Bradi2g19160.1), an acid phosphatase (Bradi2g18750.1), a glucan endo-1,3-β-glucosidase 5 precursor (Bradi2g18700.1), and a phospho-ethanolamine N-methyltransferase (Bradi2g17680.1; [90]).

**Table 3:** List of arabinoxylan-related quantitative trait loci (QTLs) reported in wheat. The QTLs on chromosome 1BL, are proposed to coincide [90,91]. From the available literature it is unclear whether the QTL reported on chromosome 3D by Nguyen et al. [92], and the meta-QTL identified by Quraishi et al. [90], on the long arm of the same chromosome coincide. Additionally, the identity of the two QTLs reported on the short arm of chromosome 6B by Quraishi et al. [90] and Lovegrove et al. [91] is unclear.

Chromosome Location	Associated Trait(s) ^1^	Explained Variance	Type of Analysis ^2^
1BL [87,90,91]	A:X of TOT-AX; A:X of WE-AX	35–42%	QTL mapping
	WE-AX extract viscosity	32–37%	QTL mapping
	WE-AX extract viscosity	≈27%	Meta-QTL analysis + GWAS validation
	RV flour aqueous extract	16–24%	QTL mapping + GWAS validation
1BS [91]	TOT-AX content	-	QTL mapping
2A [92]	TOT-AX concentration	13%	QTL mapping
3A [90]	WE-AX content	-	GWAS
3DL [90]	WE-AX extract viscosity; WE-AX content	-	Meta-QTL analysis + GWAS validation
3D [92]	TOT-AX concentration	4%	QTL mapping
4D [92]	TOT-AX concentration	19%	QTL mapping
5B [90]	A:X of TOT-AX	-	GWAS
6BS [90,91]	WE-AX extract viscosity; A:X of TOT-AX	≈58%	QTL mapping + GWAS validation
	RV flour aqueous extract	≈12	
6BL [86,90]	WE-AX extract viscosity	≈59%	QTL mapping + Meta-QTL analysis
6B [92]	TOT-AX concentration	3%	QTL mapping
7A [88,90]	A:X of TOT-AX	-	GWAS
	Dough viscosity ascribed to soluble pentosans	-	QTL mapping
7B [90]	A:X of TOT-AX	-	GWAS

^1^ Trait abbreviations: A:X = arabinose to xylose ratio; TOT-AX = total arabinoxylan; WE-AX = water-extractable arabinoxylan; RV = relative viscosity. ^2^ Analysis abbreviations: QTL = quantitative trait locus; GWAS = genome wide association study.

## 5. Challenges with the Introgression of Rye Chromatin

As discussed in Section 1, there are multiple successful examples of introgression of target rye chromatin in wheat [2,3,4]. This endeavour is mostly achieved by means of traditional breeding methods and relies on spontaneous recombination between wheat and rye chromosomes [2]. Thus, it presents two key challenges. First, the crossability of wheat with rye is under genetic control. Three major genes, *Kr1* on 5BL, *Kr2* on 5AL, and *SKr* on 5BS, are known to affect this trait in wheat [93,94,95]. In their dominant state, these genes actively repress crossability, whereas in their recessive state they are “null” alleles, implying that an extra dosage of the *kr* alleles exerts no ulterior positive effect on crossability [96]. The dominance of these alleles is incomplete, with the *Kr1* and *Kr2* loci being either complementary or additive [95,96]. The crossable phenotype occurs at high frequency in wheat landraces and varieties from Asia (i.e., China, Japan, East Siberia, and Iran), whereas Western European wheat varieties are mostly poorly crossable with rye [96]. Therefore, a necessary step to facilitate R chromatin introgressions in European wheat is the introgressions of the *kr* alleles.

Second, translocation of rye chromosome segments onto wheat chromosomes depends on the degree of homoeology between the wheat and rye chromosomes involved [2]. A comparison of the genetic maps of wheat and rye revealed overall extensive conserved collinearity [97]. These new results are in accordance with previous studies on wheat–rye homoeology as well as practical breeding experience [3,98,99]. Complete synteny is realised only between chromosome 1R and wheat chromosome group 1 [97,98,99]. Next, chromosomes 2R, 3R, 5R, and 6R are mostly syntenic with their respective wheat chromosomes groups. Nevertheless, in chromosome 6R, there is evidence for an important translocation event between the distal segment of its long arm and the corresponding distal segment of the long arm of wheat chromosome group 3 [97,98]. Lastly, the synteny between chromosomes 4R and 7R and wheat chromosome groups 4 and 7 is limited to narrow stretches of the chromosomes and there is evidence for several translocation events [97,98]. Most notably, an extensive translocation occurred between a consistent segment of the long arm of chromosome 4R and the short arm of wheat chromosome group 7; however, the short arm of chromosome 7R shows homoeology with the long arm of only wheat chromosome 4A [97,98].

These results on wheat–rye homoeology are important to consider when approaching the introgression of AX-related rye chromatin. For example, both the studies on addition lines and the most recent transcriptomic work have shown that chromosomes such as 4R, 6R, and 7R have great potential as source of AX-related genetic material [46,48,70,73]. However, the complex homoeology relationship between these chromosomes and the wheat genome can determine additional challenges for the introgression in wheat of rye chromatin from these chromosomes.

## 6. Concluding Remarks

The health-promoting effects of an adequate DF intake in general, and of AX more specifically, are well established [11,14,33]. In order to provide healthier food options to the general population, research is needed throughout the food value-chain. When considering cereal-based products, this value chain starts from the breeding of new varieties. The introgression of rye chromatin in wheat already allowed for substantial breakthroughs in wheat breeding for resistance and agronomic traits [2,3,4]. The analysis of wholemeal of wheat–rye addition lines proved that the introgression of rye chromatin can increase AX content up to the levels of the rye donor [47,48]. Thus, to address these new breeding challenges in terms of quality and nutritional aspects, rye chromatin has the potential to once again contribute significantly to wheat breeding.

## Figures and Tables

**Table 1:** List of genomic regions regulating arabinoxylan content in rye that were determined by the characterisation of wheat–rye translocation and addition lines. Chromosome 1R of *Secale cereanum* is suggested to be the product of a translocation between *S. cereale*—acting as short arm donor—and *S. montanum*—acting as long arm donor [46,47]. Chromosome 4R of *S. cereanum* is derived from *S. cereale* [46]. Chromosome 6R of *S. cereanum* is proposed to be either the product of substitutions of a *S. cereale* 6R with segments from *S. montanum*, or to originate entirely from *S. montanum* [46].

Chromosome Location	Rye Chromatin Source	Effect ^1^	Observed In
1RS [47]	*S. cereanum* (*S. cereale* × *S. montanum*)	↑ AX	Wheat–rye 1BL/1RS translocation line
1R [46]	*Secale cereanum* (*S. cereale* × *S. montanum*)	↑ TOT-AX, ↑ WE-AX	Wheat–rye disomic addition line
2R [70]	*S. cereale*	↑ Soluble DF, ↑ AX	Wheat–rye disomic addition line/centric wheat–rye translocation lines
4R [46]	*Secale cereanum* (*S. cereale* × *S. montanum*)	↑ TOT-AX, ↑ WE-AX	Wheat–rye disomic addition line
4R [47]	*S. cereale*	↑ TOT-AX, ↑ WE-AX	Wheat–rye disomic addition line
5R [70]	*S. cereale*	↑ Soluble DF, ↑ AX	Wheat–rye disomic addition/single D-substituted triticale
6RS (or proximal L) [70]	*S. cereale*	↑ Soluble DF, ↑ AX	Wheat–rye disomic addition/single D-substituted triticale
6R [46]	*S. cereanum* (*S. cereale* × *S. montanum*)	↑ TOT-AX, ↑ WE-AX	Wheat–rye disomic addition line

^1^ Effect abbreviations: AX = arabinoxylan; TOT-AX = total arabinoxylan; WE-AX = water-extractable arabinoxylan; DF = dietary fibre.

**Table 2:** Selected summary of the differentially expressed genes (DEGs), which are proposed as rye candidate genes for arabinoxylan biosynthesis in the work of Kozlova et al. [73] (please refer to the original publication for the full lists). Expression of these candidates was the highest at milk stage, and then declined at dough stage and full kernel maturity.

No. of DEGs	Chromosome Locations	Putative Gene	Protein Family	Biochemical Process
2	2R, 7R	*IRX14*	GT43	Biosynthesis of xylan backbone
5	1R, 3R, 7R	*IRX9*	GT43	
5	2R, 3R, 5R	*IRX10*	GT47 (clade D)	
2	4R, 6R	*XAT1*	GT61 (clade A)	(O)3 Arabinofuranosylmono-substitution
1	3R	*BAHD01*	BAHD	Feruloylation

## Data Availability

Not applicable.
